# Directed content analysis: A life course approach to understanding the impacts of the COVID-19 pandemic with implications for public health and social service policy

**DOI:** 10.1371/journal.pone.0278240

**Published:** 2022-12-16

**Authors:** Eva Purkey, Imaan Bayoumi, Colleen M. Davison, Autumn Watson

**Affiliations:** 1 Queen’s University Department of Family Medicine, Kingston, Ontario, Canada; 2 Queen’s University Public Health Sciences, Kingston, Ontario, Canada; 3 Indigenous Diabetes Health Circle, Curve Lake First Nation, Ontario, Canada; The University of Manchester Division of Psychology and Mental Health, UNITED KINGDOM

## Abstract

**Background:**

The COVID-19 pandemic has had broad impacts on individuals, families and communities which will continue to require multidimensional responses from service providers, program developers, and policy makers.

**Objectives:**

The purpose of this study was to use Life Course theory to understand and imagine public health and policy responses to the multiple and varied impacts of the COVID-19 pandemic on different groups.

**Methods:**

“The Cost of COVID-19” was a research study carried out in Kingston, Frontenac, Lennox and Addington counties in South Eastern Ontario, Canada, between June and December 2020. Data included 210 micronarrative stories collected from community members, and 31 in-depth interviews with health and social service providers. Data were analyzed using directed content analysis to explore the fit between data and the constructs of Life Course theory.

**Results:**

Social pathways were significantly disrupted by changes to education and employment, as well as changes to roles which further altered anticipated pathways. Transitions were by and large missed, creating a sense of loss. While some respondents articulated positive turning points, most of the turning points reported were negative, including fundamental changes to relationships, family structure, education, and employment with lifelong implications. Participants’ trajectories varied based on principles including when they occurred in their lifespan, the amount of agency they felt or did not feel over circumstances, where they lived (rural versus urban), what else was going on in their lives at the time the pandemic struck, how their lives were connected with others, as well as how the pandemic impacted the lives of those dear to them. An additional principle, that of Culture, was felt to be missing from the Life Course theory as currently outlined.

**Conclusions:**

A Life Course analysis may improve our understanding of the multidimensional long-term impacts of the COVID-19 pandemic and associated public health countermeasures. This analysis could help us to anticipate services that will require development, training, and funding to support the recovery of those who have been particularly affected. Resources needed will include education, mental health and job creation supports, as well as programs that support the development of individual and community agency.

## Background

The COVID-19 pandemic has affected individuals, families, communities and countries. Relationships within and between countries have been altered as illustrated by restrictions on international travel, changes to foreign aid, and varying degrees of international collaboration on disease surveillance and vaccine development and distribution. Public and private institutions (health and social service agencies, the education system, among others) have had to change and adapt dramatically. Communities have had to reconfigure how members interact, how they support each other, and the care they provide to the most vulnerable. Families and communities have been divided by geographic distance and in some cases by ideology. Individuals have seen fundamental changes to their ability to socialize, to work, to attend school, and to feel safe in their environment [1–4]. While some Canadian political and public health messaging has emphasized that we are “all in this together”, this has in fact not been the case, with very different impacts being felt by different groups, varying by socioeconomic status, gender, race, geography, ability, political inclination or age among others [3]. Differences in the experience of the pandemic include disproportionate infection rates as well as mortality in neighborhoods with high proportions of racialized people, as well as those with material deprivation and low income [5,6]. Other differences include the disproportionate impact of the restriction in services on people living with intellectual and developmental disabilities [7].

The breadth of the impact of the COVID-19 pandemic is unlike anything seen in a generation. The disruption it has caused to all aspects of individual, family, community and social life and development echo the impacts of Spanish Influenza in 1918, including the politicization of a public health response, disproportionate impact on minority groups, and scapegoating of certain groups with respect to origin and spread of the disease [8], however important differences between these pandemics include the speed of global communication, and the scientific response with respect to vaccination and supportive medical care. Many academics and policy makers have spoken about the impacts of secondary pandemics in the context of the COVID-19 pandemic, for instance pandemics of child abuse and neglect, of mental health disorders, of loneliness and isolation, of widening gaps in education and others [1,2,4,9]. Recovery from the ongoing acute, infectious pandemic, requires imagining a way forward through the pervasive impacts of COVID-19, while avoiding simplistic or reductionist thinking and policy making. There is an opportunity to consider the interconnections, the width and breadth of the human experience as societies creatively imagine what recovery will be. We believe that Life Course theory can be useful when understanding the impacts of social upheaval, such as the COVID-19 pandemic, particularly when these upheavals pervasively affect all or many components of individual, family, social and cultural life [10].

## Life course theory

Life course theory is a theoretical orientation pioneered by Glen H Elder Jr that situates people within a historical moment in time and place, in the context of specific social institutions, bound by certain normative social and cultural pathways [11]. While it takes into account age, it goes beyond a lifespan approach to contextualize age cohorts in a specific historical and social time and place.

This theory has been used to understand large social changes such as the impacts of the Great Depression [12] or the Chinese Cultural Revolution [13], as well as seemingly less sweepingly transformative and more individual social phenomena such as criminality and teen pregnancy [14,15]. Because Life Course theory seeks to explore the individual, family, social and cultural impacts of experiences from a complex and multifaceted perspective, this theory may be helpful in understanding both the immediate, short term consequences of the pandemic, as well as the long-term impacts that are likely to be experienced throughout the entire lives of individuals and societies affected. Life Course theory is not familiar to many public health practitioners, andoutlining the effects of the pandemic in this way can provide a novel and useful paradigm with which to move forward towards pandemic recovery.

The constructs of Life Course theory can be divided into three groups: Constructs related to timing (cohort or period effects), those related to trajectories (social pathways, transitions, and turning points), and then broader principles through which any impactful event can be examined (life span development, agency, time and place, timing, and linked lives). These constructs are described below [11].

**Timing** constructs include the cohort effect and the period effect. The *cohort effect* is the phenomenon by which certain historical events differentiate the lives of successive birth cohorts (for example, children born during the great depression, or young men coming of age during the Vietnam war), while a *period effect* arises when a particular event occurs over a longer time, producing relatively similar impact on a number of successive birth cohorts. This paper considers the cohort effect as the primary construct underlying the experience of COVID-19.

The three constructs in the **trajectories** domain include *social pathways*, referring to expected sequence of life stages and events. These will vary depending on cultural influences, and over time and place, but can be altered by historical forces that are outside the control of the individual (recessions, differential funding for higher education, civil or political unrest). *Transitions* refer to changes in role that make up trajectories. Graduating from school, moving out of the family home, starting a job, having a first child, and retirement, would all be transitions, which happen at different chronological ages, and the timing of which will affect one’s life course and trajectory. Finally, *turning points* are major events that produce a substantial change in the direction of one’s life.

All of these constructs can be viewed through the lens of **five principles**. (1) *Life span development* is the principle by which we view individuals in a constant state of development. Development does not only happen to young children but is occurring at every point in the life cycle. Any major historical event occurring at any point in the life of an individual, will have an impact on their ongoing development including how they assess and build physical, social, spiritual, and social abilities and goals (2). The principle of *agency* [16] articulates the ways in which people are active in making choices that impact their life and life course. Agency is a complex principle that includes concrete constructs such as self-efficacy, but also more philosophical notions of freedom and free will. Fundamentally, it proposes that people construct their own life course by making choices in the context of opportunities and constraints presented to them [11]. (3) The principle of *time and place* proposes that life courses are impacted by geographic and historical events. The same historical event will have different impact in different geographic locations. (4) The principle of *timing* implies that an event will have differential impact based on how it is timed in a person’s life. Family poverty and food deprivation will be different when experienced by a toddler than when experienced by a responsible adult caregiver. (5) Finally, the principle of *linked lives* refers to the fact that we do not live our lives in isolation, and that our life courses are profoundly shaped by our social networks, our culture, and by the impact of events on the lives of the people around us.

In using Life Course theory to explore the impact of the COVID-19 pandemic and public health countermeasures, this paper seeks to illustrate the complexities of the impact of the pandemic on many aspects of people’s lives, as well as the implications these complex impacts will have for health, social and economic policy and recovery.

## Methods

### Setting

The data used for this analysis is data from “The Cost of COVID-19” research study carried out in Kingston, Frontenac, Lennox and Addington counties (KFL&A) in South Eastern Ontario. The data for this analysis was collected between June and December 2020 and includes two data sources described below. KFL&A is an area with a population of roughly 210,000 people [17] that was relatively spared during the first year of the pandemic, with only 758 cases of COVID-19 and a single death [18,19]. The region had a strong public health response, with strong leadership and excellent communication strategies, and high levels of compliance with public health guidelines. As elsewhere in Ontario, primary and secondary schools were closed to in person learning from March 2020 to September 2020, and reopened for masked, in person learning until the Christmas break in December 2020, at which point there was a second period of school closures [20]. Following a strict restriction of gathering size at the onset of the pandemic, people were allowed to gather in larger groups from June to September, when gatherings were again restricted following a rise in cases and remained tightly restricted into early 2021 [20]. An initial period of extensive closures, including of indoor and outdoor play spaces, parks, and recreational activities, was followed by a gradual and selective reopening of services by the summer of 2020 [20] but people were still being encourage to avoid common spaces and to physically distance as much as possible. With respect to economic support, between March and September 2020, the Federal government implemented the Canada Emergency Response Benefit (CERB)–a benefit providing people with 500$CDN per week for lost income caused by the pandemic [21]. This benefit required people to have had a minimum income in 2019 to be eligible.

### Ethics

This study was reviewed and approved by Queen’s University Health Sciences and Affiliated Teaching Hospitals Research Ethics Board. All participants provided informed written or recorded consent to participate in this study. All methods were performed in accordance with the relevant guidelines and regulations. In addition to this ethics board, “The Cost of COVID-19” study was planned and conducted with support of an oversight committee from the Indigenous Health Council to ensure that the design, data management, and dissemination of findings were consistent with the principles of OCAP (Ownership, Control, Access and Possession) [22]. AW (author) was hired as an Indigenous Research Assistant to support communication with the oversight committee and outreach to the Indigenous Community.

### Spryng.io micronarratives

The first data source was participant-offered short stories, or micronarratives, collected between June 30^th^ and November 3^rd^ 2020 using the Spryng.io platform [19], a technology that allows participants to type or upload an audio story using a laptop, tablet or phone in response to one of three pre-programmed prompts (ex: “Please tell a story about the worst OR best impact of COVID-19 on you and/or your household”). Participants then interpreted their own story by plotting responses to a series of pre-defined questions, and finally to respond to certain demographic questions anonymously. These micronarratives and accompanying responses were uploaded to a secure server and transcripts downloaded. In this study stories ranged in length from an average of approximately 150 to 800 words, with most in the 250–500 word range. Respondents to this portion of the study were adults 16 years of age or older. Sampling was in the first instance opportunistic: the online data collection tool was distributed through individual networks, social media, and partner organizations. Second, intentional sampling was performed with certain groups, namely urban Indigenous People, people living in poverty, and people experiencing homelessness. The purpose of this intentional sampling was to ensure representation of segments of our population who are often underrepresented in such studies due to inability to access the study platforms (eg lack of technology or technological literacy) and/or lack of trust in research processes, among others [23,24]. Recruitment continued until 200 participants had been involved. This number was chosen due to sampling recommendations for quantitative analyses in Spryng.io work, which are not applicable to the qualitative analysis completed here. Two research assistants actively recruited respondents outside an integrated care hub established in Kingston as a response to homelessness during the COVID-19 crisis, as well as participants attending cultural events held by the local Indigenous community. The research assistants also attended two local food programs serving people experiencing food insecurity.

### Key informant interviews

The second data source used for this analysis was in-depth interviews with key informants recruited from organizations providing healthcare and/or a variety of social services, including child welfare services, school support, sexual assault and domestic violence services, and mental health services among others. These interviews served to triangulate the responses from individual first-person micronarratives by asking interview participants to reflect more broadly on the experiences of individuals in the groups they served as well as within their own organization. Participants from organizations providing services to Indigenous People and other marginalized groups often excluded from research were intentionally recruited for the reasons mentioned above. Sampling of key informants was intentional. Participants were recruited by phone or email. Questions included populations served and services provided, as well as questions in five main themes: (1) impact of the pandemic on mental health; (2) impact of the pandemic on family and partner conflict; (3) impact on access to services; (4) service adaptations; and (5) ideas for future adaptations. Participants were provided space and time for open ended answers, and to add anything they felt was important and not covered by the questionnaire. Interviews were completed by trained Research Assistants via Zoom to adhere to public health guidelines, audio recorded, and transcribed verbatim. Interviews were completed between October and December 2020 and participants were recruited until saturation of themes was reached. Interviews ranged from approximately 45 minutes to 90 minutes in duration. While these interviews concluded slightly later than the micronarrative collection, the local pandemic situation (infection rates, public health countermeasures) was essentially the same throughout that time.

### Data analysis

Data was analyzed using directed content analysis. In directed content analysis, a framework with key concepts or constructs is identified to create initial coding categories. These must be clearly defined (as has been outlined above for Life Course constructs). Transcripts are read and coded to these existing codes, with text that cannot be categorized into the existing coding framework is given new codes, if appropriate, or assigned to sub-categories of existing codes. In this way, previous theoretical frameworks can be tested against new data, and possibly expanded and strengthened. Likewise, new data can be understood through the lens of existing theoretical frameworks, which can help expand the use and actionability of findings [25,26]. For the purposes of this analysis, transcripts were reviewed and coded to the core Life Course theory constructs [11,25,26].

Both sets of transcripts were reviewed in their entirety by four researchers on the research team (EP, CD, AW, IB) to identify emerging themes that warranted further investigation and analysis. EP is a qualitative researcher and clinician whose teaching, clinical care and research focus on equity deserving groups and the impact of trauma and adversity on health. CD is a public health scientist and mixed methods researcher whose research interests include children and youth. AW is a community based Indigenous researcher. IB is a predominantly quantitative researcher and clinician whose research focuses on child health. Emerging themes included complex experiences of COVID-19 and public health countermeasures that appeared to far outweigh the impact of the virus. Life Course theory was identified as a theory with sufficient complexity to illustrate many of the impacts of the COVID-19 pandemic and associated countermeasures. One researcher (EP) used Directed Content Analysis [26,27] to code the entire data set using the eight Life Course constructs outlined above as primary categories or codes. Data that could not fit well into existing Life Course constructs was coded separately. Following the initial coding by EP, the analysis was brought back to the three other researchers (IB, CD, AW) who, having already read the transcripts in detail, reviewed the coding process and themes for reliability and consistency.

## Results

### Demographics

Table 1 provides information on key characteristics of the respondents for the Spryng.io micronarratives. While the majority of respondents were female, there was a broad age range and income distribution. Participants were given the opportunity to self-identify as Indigenous. Other specific ethnic backgrounds were not identified.

**Table 1:** Description of the sample providing Spryng.io micronarratives and sociodemographic information.

Variable	Category	n = 210 (%)
Gender	Female: Male: Other/prefer not to answer:	158 (75) 47 (22) 5 (2)
Age	16–21: 22–34: 35–49: 50–64: 65–79: Older/prefer not to answer:	12 (6) 57 (27) 55 (26) 59 (28) 23 (10) 4 (2)
Income	10,000–39,000: 40,000–79,999: 80,000–124,999: >125,000 Prefer not to say:	62 (30) 41 (20) 47 (22) 32 (15) 28 (13)
Ethnicity/Race	Indigenous Visible Minority (Other)	28 (13) 3 (1.4)
Where do you live	KLF&A: Ontario (other): Outside of Ontario:	176 (84) 28 (13) 6 (3)

Thirty one service providers or volunteers participated in the in-depth interviews (see Table 2), including fifteen who identified their primary target population as Indigenous Peoples. Sixteen of the organizations had a more general mandate. The organizations provided a broad variety of services, including primary care, mental health for children and adults, sexual assault and domestic violence services, shelter services, child and family services, school supports, and supports for people living with disabilities, among others. Respondents were evenly split between service providers and administrators and ranged in experience from 6 months to 20 years with their organization. Some interview participants had held many roles within their organization and community. Organizations provided a variety of types of service delivery (phone, drop in, in person appointments, groups, home visits, etc) and significant adaptations had been made to all services since the start of the pandemic.

**Table 2:** Description of the sample of service providers who participated in the in-depth interviews.

Variable	Category	Number N = 31 (%)
Target population	Urban (exclusively) Rural (exclusively) Both	8 (26) 0 (0) 23 (74)
Service sector*	Health care services Housing/Shelter Education Social services/other	9 (29) 2 (6) 3 (10) 17 (55)
Role in organizations	Admin/management: Direct service provision: Volunteer:	12 (39) 14 (45) 5 (16)
Length of time in organization	<2 yrs 2–5 yrs 5–10 yrs >10 yrs	4 (13) 12 (39) 6 (19) 9 (29)

*note that there is overlap between sectors; organizations have been categorized based on primary mandate.

Due to the qualitative nature of this analysis, inter-group differences were not specifically quantified or compared, however the themes that emerged were consistent between the first-person micronarratives and the in-depth service provider interviews.

### Directed content analysis: Life course theoretical framework

The majority of data fit meaningfully into the constructs of the Life Course Framework. Secondary codes under each construct included whether the experience reported by the participant with positive or negative with respect to this construct. Most of the data reported negative impacts of the pandemic on the constructs in question, however these variances are discussed below. The only theme in our data that did not meaningfully fit into the constructs of Life Course Framework was related to the impact of culture on Life Course. This was particularly salient in our findings due to our sampling of Indigenous people. While many participant quotes related to culture could fit under other Life Course constructs such as transitions, there seemed to be an overall theme of importance of culture that was inadequately addressed by existing constructs and such an additional theme was developed.

### Trajectories

#### Social pathways

Respondents illustrated ways in which their social pathways have been disrupted since the onset of the pandemic. People’s trajectories were actively disrupted by the public health measures put in place in response to the pandemic, including disruptions to education or employment, such as delaying entry to university, disengaging from an academic program partway, or to being unable to find work.

*I am on [Ontario Disability Support Program]*. *I want to get a job but so many businesses have closed down and they’re not willing to start businesses due to worry about COVID. Been on [Ontario Disability Support Program] since August. I’d love to go back to work but I can’t. (Spryng 5654)*

*My summer job was cancelled*, *and despite applying at several other places*, *I am unemployed*. *I have had a job since grade 10 of high school*, *working all summer every summer*. *Being home has made me feel very lonely*. *(Spryng 4051*)

*I also have several [First Nations*, *Inuit*, *Metis] students who have moved from their home territories thinking that they are going to be doing school in-person*. *So I have folks from like northern communities um and from like not Ontario*. *And they came here*, *thinking that they would be in a cohort*. *And now they’re sitting by themselves*, *in an apartment*, *in the south*, *and they don’t know anyone*. *(6A*)

There were also significant disruptions to roles, such as students having to become caregivers, and parents who felt they were no longer able to effectively provide for their children due to new poverty or food insecurity.

*And uh so what became really evident is how many children in the middle class of the demographic were accessing that fresh fruit and vegetables through the Food Sharing Program and through [program*].

*So now we have a whole section of our community that has not had to access Food Banks*, *has not had to access support from Salvation Army*, *has not had to go to churches*, *didn’t know the network to connect themselves to bridge that gap that they were experiencing in their own reality of what this pandemic looked like*. *(9A*)

*That’s how people felt*, *is like*, *I’m failing as parent*. *[…] People were afraid they were going to lose their children to Children’s Aid because they didn’t have food to feed [them]*. *And that is going to carry with them for a long time [and] we’re not ever going to see that*. *(9A*)

Some of the impacts of the pandemic on social pathways were unique based on cultural groups, with Indigenous People and newcomers more likely to be identified as having new obligations to family members due to the disruptions of the pandemic [28]. Some participants did report positive change, such as improvements in income through access to government pandemic relief programs, delayed evictions, and reprioritization of personal and family time. While the initial disruptions were viewed as a crisis and responded to as such, as the pandemic lingered, social pathways became more and more disrupted with implications for people on identity, sense of agency (see below), and choices people made about employment and education, among others.

### Transitions

The impact of the COVID-19 pandemic on transitions was very clear from our respondents and many kinds of transitions were profoundly affected. People described the isolation of being a first-time parent, without access to friends or family members to mark special or difficult times. Others spoke of missing key symbolic moments which are recognized as meaningful across cultures and throughout time: graduation ceremonies, birthdays, funerals, and other rites of passage. These ceremonies anchor us as social beings within our communities, and the long-term impact of missing them should not be underestimated.

*Pregnancy can be very lonely even if you are surrounded by the most supportive husband and family—and being pregnant during COVID was extra lonely (Spryng 5876*)

*After competing my course work in late April*, *I was now a university graduate*, *but I do not feel like it*. *I feel that [university name] has made VERY little effort to celebrate their graduates*, *especially compared to other universities and high schools*. *My parents made no effort to celebrate my accomplishment*, *and my mental health was affected as I felt my past four years of hard work has gone uncelebrated*. *(Spryng 4051*)

*I realize that there was no way around the circumstances caused by the pandemic but the combination of quarantine*, *self-isolation and grieving is brutal*. *(Spryng 3962*)

People also described more subtle but equally important role transitions such as youth transitioning out of family and children’s services and being unable to find meaningful work (or any work at all) during their transition to adulthood.

*So our youth that have aged out [of the child welfare system] and are not in school*. *They have no supports*. *We do work with some of those youths that have aged out of [care] and are no longer sort of under their control*. *And yeah this this whole pandemic and not being able to access the services and the places that they would normally go to*, *connect to WIFI or to*, *you know*, *to go in and get out of the heat or out of the cold*, *whatever the case may be*, *it’s all been taken away*, *right*. *Um so seeing a lot of increased drug use*, *increased risky drug use um and unfortunately with a few deaths in town*, *directly related to that*. *[…] I mean they’re just starting out kind of their adult life*, *so to speak*. *And then everything shuts down and they have nowhere to go and no contacts*, *no social life*. *And I think that’s been the hardest for them*. *(4A*)

Indigenous students who had enrolled in Indigenous-specific higher education programs and support services to explore their Indigenous identity and to transition to the role of teacher lost out on mentorship opportunities to help them with these transitions.

*For some of our students who now don’t step foot in the college*, *you know*, *[*..*] the reality is that we could have students that get a certificate or a diploma without ever stepping foot on our campus*. *And if that’s the case*, *they kind of missed that opportunity to reconnect with their culture [to] explore some of that and then continue to educate their children*, *other people in the community*, *their families*. *And so I feel like we’re missing*, *you know*, *this really important group of people who could then continue and of course we’re looking into*, *you know*, *reconciliation in trying to get people to take action*. *(10A*)

Parents transitioned to the role of grandparents without being able to meet their grandchildren. While some people were able to see the silver lining of how the pandemic had affected transitions in their lives (longer parental leaves, witnessing key milestones in children that they may have otherwise missed due to being at work), overall, respondents experience of the impact of the pandemic on transitions was negative.

### Turning points

**]**COVID-19 itself may be looked back on as a turning point for many: a moment when lives changed direction, deviating from previously set social pathways. Turning points ultimately must be viewed retrospectively, since by definition they would be times one would look back on as key moments of change. Nevertheless, our respondents identified life events which seem likely to be looked back on as turning points in the future. These included divorces and separations of long-term partners, first experiences of homelessness, and deaths of loved ones (in our responses there were no direct experiences of deaths from COVID-19, but respondents reported other deaths including from drug overdose and homicide in the context of intimate partner violence).

*The Covid lockdowns came during the midst of my divorce*. *I believe it escalated it into a higher conflict situation (Spryng 5791*)

*There’s another kid who is also 16 and his dad kicked him out because he was smoking weed*, *which is not uncommon for a 16-year-old*, *especially in that area*. *[*..*] So this one boy*, *who was you know caught with the marijuana*, *he doesn’t want to go home anymore*. *And they don’t want him home*. *So the youth counsellor had been working with them and they just decided no that’s done*, *we don’t want to pursue that anymore*. *So we got transitional housing options for him*. *(15B*)

*Covid has left me and my boyfriend homeless*. *He then died from fentanyl because of all the covid stress*. *(Spryng 5675*)

*So we’ve already seen um*, *you know*, *domestic violence that has led to murder in during the pandemic*.

(*9B*)

Long term, potentially irreversible, effects on the health and education of children were also mentioned, including dropping out of high school, becoming obese, or experiencing new or acute material deprivation. Older students failed to complete or opted to defer their plans for higher education.

*Parents that don’t have a lot of resources*, *have decided to keep their kids home*, *they’re sedentary all day*. *So they’re on the screen or they’re watching TV*, *they’re gaining weight*, *and all this is becoming entrenched*. *And so*, *even if everything went back to normal tomorrow–the child that has gained 20 or 30 pounds during COVID*, *that’s a life-long*. *Now that that child has obesity*, *that doesn’t go away*, *right*. *That’s a forever problem that’s type II diabetes as a teenager problem*. *It’s significant*, *because even if all the restrictions were lifted tomorrow*, *which they won’t be*, *even if they were*, *these new pattern of increased substance abuse and sedentariness have long-lasting consequences*. *[…] And so I worry about my kids that were starting grade 9 and grade 10 –this could be the end of high school for them*. *If they fall behind in enough courses […]*, *they’re not going to catch up and they’re not going to be motivated to*. *(5B*)

While the vast majority of the turning points identified were negative, a few did report that the respite provided by the Canadian Emergency Response Benefit allowed them to reconsider their employment and move their lives in a new direction.

*While I was receiving CERB [Canadian Emergency Response Benefit] I had the time to start my own business without worrying to much about what would happen if I wasn’t immediately successful*. *Starting my own business had been something I wanted to do for a while and it was a great experience and took off relatively quickly*. *(Spryng 5910*)

Some respondents identified these turning points as being directly related to the pandemic and associated countermeasures, while this was not clear in other cases. In all cases, the pandemic affected people’s choices and strategies in how they managed or coped with the turning points they experienced.

### Principles

Five core principles have been described within the Life Course theory that allow us to better understand the trajectory components of the theory in the context of a cohort effect. These principles can help us to understand how and why some people do better or worse given superficially similar circumstances. Our data highlighted these principles, as outlined below.

### Life-span development

Our data illustrated how the COVID-19 pandemic and associated public health countermeasures impacted people across the lifespan. Participants’ responses tended to focus on children and the elderly, but nevertheless illustrated the constant evolution and change experienced by human beings, particularly in response to the unusual circumstances of the pandemic.

*My child is now 15 months old and has never played with another child*. *(Spryng 5797*)

*One heart breaking change I have seen is that our 3 year old now knows to give people space*. *We were playing on the sidewalk outside of our house when a neighbor family was walking down the street our daughter yelled “PEOPLE*!*” Got up and ran to the other side of our yard*. *(Spryng 4114*)

*Oh for some of our Elders*, *[…] I know this directly from individuals affected that with social distancing it means that there is a real reduction in physical activity*. *Like profound reduction (13A*)

While many of the perceived changes were negative, there were positives as well particularly for people who were able to improve their access to and ease with technology as a means to reach out to the world and learn new things through platforms that were previously unavailable to them.

*The best impact of Covid-19 on my household (me) is that as an independent*, *disabled senior on oxygen the world has opened up to me again through the computer*. *Instead of loading up the car with my oxygen equipment and walker and going to the gym*, *my trainers now come to me through zoom*. *Similarly*, *I now can attend political meetings*, *church*, *doctor’s appointments*, *interest courses*, *even concerts through the use of technology without having to expend effort and money trying to get from point A to point B*. *(Spryng 5561*)

### Agency

Agency, or the lack thereof, was perhaps one of the most starkly differentiated themes in our data. Findings ranged from people who obviously felt a significant sense of agency and/or who witnessed agency, strength and resilience in others, to many who felt completely powerless in the face of the pandemic.

Whether or not people experienced a sense of agency seemed to have a particularly important impact on their overall experience. While some quotes identify a sense of strength, self-efficacy and resilience:

*Man*, *like when families work together they’re unstoppable*. *Like we need to encourage that*. *(2A*)

So there’s been challenges, you know, certainly there’s been challenges, but at the same time it’s been so much creativity that has been born, you know because you’ve got to make it work. (3A)

Others echo fear and insecurity related to profound sense of fear and lack of control:

*The beginning of the pandemic was the worst*. *I didn’t know what we were up against*, *a lot of unknowns*. *I was nervously eating a lot of bread & potatoes & foods that aren’t good for me*. *I’d almost be falling asleep at night & experience anxiety*, *fear etc*. *(Spryng 5568*)

*I think they’ve settled into some kind of sense that*, *well this is my life and this is how it’s going to be*. *And sort of in a dark place where we have no hope that it’s going to change*. *(11A*)

*And they don’t seem to have the resilience on their own to find a way to fill that gap*. *I suspect a lot of them are just watching tv or playing video games*. *(11A*)

For instance, some new mothers clearly felt disempowered, struggling with isolation, loss related to lack of social and family support, missing out on celebrations and ceremony related to their child’s birth and development. Others viewed the same situation as positive–time to connect with their co-parent, an opportunity to demonstrate strength in “getting through this” as their small nuclear family.

*I feel like myself and my spouse have been a good team*. *Our kids are happy and healthy*, *active without much restriction (hikes*, *backyard play*, *themed learning weeks based on child-lead interests*, *etc)*. *Giving them positive memories and as little worry as possible regarding Covid-19 is a top priority to us*. *(Spryng 4202*)

Agency cannot be viewed in isolation. Whether one has a sense of agency will depend in part on other pre-existing conditions such as financial and material security, presence of a supportive or abusive partner or social network, pre-existing mental health, social isolation, and cultural grounding, to name a few.

*It’s about loss of control*, *which is often an impact of sexual violence in itself*, *right*. *(6B*)

### Time and place

Time and place was the least explicitly addressed aspect of the life course model in our study data. Since the majority of our respondents were from the Kingston area, we did not have a wide geographic diversity. In our sample, the variable impacts of the public health countermeasures on time and space were illustrated primarily in the urban-rural divide, and then specifically for people living in remote Indigenous communities.

*All of the webinars and stuff that we’ve been offering were then not accessible to the indigenous people that didn’t have the technology to be able to listen in on those webinars*. *[…] In our [University Program] the lack of good internet or internet at all because we were going to open a new [program] in [remote community name]*, *like over half of the students were living in communities that had dial-up*. *And you can’t do Zoom and those things with the dialup*. *(12A*)

*I would add rural communities to that because for rural people*, *services are few and far between to begin with*. *And then when you start taking away resources like maybe they’re not making as much in their job*. *Maybe they’re not*, *you know they can’t travel to services now*. *And then maybe the rural services don’t have the enough of the digital virtual stuff going on that they could provide virtual support*. *(2A*)

*I know we had one situation where we had some equipment that was supposed to go to a child up North and the family originally would have been coming down and would have taken it home with them*, *after an appointment*. *They weren’t coming down so the child’s been without the equipment*. *(2B*)

The majority of our respondents were from KFL&A, a region in southeastern Ontario with both urban and rural communities, and with relatively low COVID-19 prevalence. While participants reported a great deal of fear, as well as a great deal of self-imposed restriction on their activities in addition to the restrictions imposed by public health authorities, no one in our study reported the loss of, or even infection of a close friend or family member from COVID-19, something that would have been very different in another geographic location, or even in KFL&A at a different point in the pandemic.

### Timing

Similar to life-span development, our data illustrated the differential impact of the pandemic depending on the timing in which it occurred in a persons’ life. More than just a age related construct (ie., this happened to me when I was X years old), respondents described how the intersection of the pandemic with a certain moment in a person’s life (for instance, the impact of a pandemic lockdown that occurred at the same time as an evolving custody battle and change in employment, or the impact of elementary school closures on a mature student trying to complete University studies while being a single parent) would significantly influence the impact the events had on life course, being more significant for some, and less for others, depending on the point of intersection. Generally, our respondents illustrated greatest concern about the impact of the pandemic on the social, emotional, and educational lives of developing children and youth.

" *kids are suffering" they are not getting the interaction that they need on so many different levels*. *Emotional*, *mentally*, *physical contact with family and their friends*. *[…] Kids are starting to worry about school*. *The 6 year old is afraid her friends won’t know her or she won’t know them with masks on*. *The boys are afraid of their backpacks and coats on their chairs will be too close*. *They won’t be able to play the same games*, *there won’t be hockey or all fun with their friends*, *the 13 year is just afraid all together*. *(Spryng 5117)*

*We’re seeing teens with um depression, anxiety, social isolation that’s getting worse*. *We’re seeing lots of kids that are really, I’m really worried about how they’re falling behind in school*. *They may have had special needs or individualized education plans, but now parents have decided to keep them home probably based on not accurate health information. And now the kids are not able to receive the specialized supports that they would have needed. And I’m worried that they’re falling farther and farther behind. […] And I worry that even when this is all over, this kind of stuff is going to be long-term for these kids. And that, that’s the part that I that, like I said, that’s the part that is keeping me awake at night, it’s the kids. (5B)*

### Linked lives

Finally, the principle of linked lives was vividly illustrated throughout our findings. On the positive side, participants identified increased time spent with loved ones and household members as a benefit of the pandemic.

*It has strengthened our relationships in a way that can never be replicated (Spryng 5769*)

*This crisis has allowed me more time to realize that family*, *love*, *and friendship are the most important things in life and so much time spend online is a colossal waste of energy (Spryng 5775*)

On the negative side, previously fragile or tense relationships exploded under the added strain of forced social isolation. Front line workers identified as a main source of stress the risk of bringing infection home to their loved ones, again illustrating the impact of linked lives. Workers who could not work from home observed others spending more time with family in seemingly harmonious activities felt guilty or sad that this could not be available to their own families. And people who were alone were lonelier, in some cases perhaps, than ever before.

*I feel guilty when I come into work that I’m not home with my kids*. *I cry on my way into work as I watch other families out together on bikes*, *going for walks*, *just being together*. *I feel guilty that we’re not able to have that time together*. *(Spryng 4152*)

*Covid-19 has impacted my household in a negative way*, *as it has significantly increased the tension in my house*. *[…] Since we have been at home for the past couple of months my parents have been fighting more*, *my parents have been on edge and taken their anger out on us kids*, *and my siblings and I have been getting into more disagreements*. *(Spryng 4050*)

*But the depth of this isolation*, *even if it is sporadic*, *I genuinely don’t think that we’re going to understand it*. *We’ve*, *we’ve never gone through this kind of isolation*, *not really*. *And that’s a blanket statement that applies to Canada and essentially the world*. *(8A*)

Clearly, stresses and disruptions in trajectories felt by one family member often had significant impact on others. This is powerfully illustrated by a quote in which a child illustrates the impact that new food insecurity has had on his mother in what he says to a worker bringing them fresh fruit:

*And then the children*, *when they would say–“I’m thankful that you’re here*, *my mom’s not going to cry today”*. *(9A*)

### Additional theme: Culture

While most of the themes emerging around culture could fit in original Life Course constructs, gaps remained in capturing culture as core principle that affects a person’s life course, not completely independently, but still distinct from the original constructs. In our data, the theme of culture emerged predominantly around Indigenous culture, with the importance of cultural belonging, spaces for engaging in cultural practices, and differential impacts of pandemic restrictions depending on core components of cultural practice emerging. Additional culture-related themes involved newcomers to Canada who articulated a cultural expectation that they would provide care for their extended families. While all the quotes in this section relate to Indigenous culture, we anticipate that a similar study done in a different setting, with more cultural diversity, would uncover the importance of culture for other groups as well.

*It was really humbling to see finally some indigenous families not having to be in the colonial structure of schools and school boards*. *That they were connecting to the land*. *They were including their children in the baking*. *They were including their children in the measuring*. *They were they were going back to our traditional ways of how we teach our young*. *And it wasn’t the western reading*, *writing*, *arithmetic*. *It was in the most holistic way*. *So if we’re going to take something good out of it*. *(9A*)

*And to just have that level of care*, *to know that we just have to keep going*. *You know*, *I just think it really speaks to who we are as indigenous people*. *We’re resilient*. *We can get through anything*. *(3A*)

*From a cultural perspective*… *I think our ways of coming together have been totally*…*constrained*. *So*, *and I think now people are trying to find ways of doing that*, *so trying to figure out ways*. *So I’m starting to hear of things like people are doing little social distance things to try and reconnect*. *So I heard about a Moon Ceremony recently*. *A little social distance Moon Ceremony*. *There’s been social distance Water ceremonies*. *There’s going to be a social distance Sacred fire*. *(13A*)

## Discussion

Our findings illustrate that the pervasive impacts of the COVID-19 pandemic and associated countermeasures echo the Life Course constructs as described by Elde [11]. We believe that this framework provides an important lens in understanding how to consider the complex and interrelated Life Course constructs when planning for recovery. The strength of Life Course theory as applied to the COVID-19 pandemic is that it paints a complex and nuanced picture of how the pandemic has affected the lives of individuals that may fundamentally alter their life trajectories in the short, medium, and likely long term ways. Learning from past major events which have been analyzed through the lens of Life Course theory, we believe that this analysis can allow program developers, service providers, and policy makers to think about how and which of these constructs need to be addressed by different initiatives as we rebuild.

Life Course theory-based analyses have demonstrated the long term impacts of major disruptions on peoples’ lives, such as that of the Great Depression on children in the United States [12], as well as the impact of the Cultural Revolution in China [29]. These studies, completed long after the fact, have shown how major disruptions can have impacts over the entire lifespan, both due to the initial insult, but also due to the pathway of cumulative advantage or disadvantage triggered by the initial event. Emerging studies are beginning to look at Life Course impacts of COVID-19 pandemic, though they are limited by the timeframe which they are studying. A German study exploring adolescent well-being and transitions to post-secondary education [30] explored the impact of the pandemic on mental health, educational and career plans. A second identified the importance of social relationships and coping skills on adult quality of life [31] and made recommendations to mitigate the effects of these for future pandemics. A third study in pre-publication explores the concept of self-efficacy and valence as it pertains to life satisfaction [32]. These constructs align closely with that of Agency. These studies will be necessarily have a near-sighted view of the impacts of the pandemic, but will add to the call to reflect on how these constructs, and other Life Course constructs as defined by Elder, will shape people’s lives, and therefore society, going forward, and how they can be leveraged both to recover from the current pandemic, and to prepare for other pandemics or major events which will inevitably occur.

A construct which we believe is missing from Life Course theory and that was important in understanding the experience of our participants is that of culture. Culture was a strength for our study participants, which is consistent with other publications exploring the experience of Canadian Indigenous culture during COVID-19 [33,34]. We know however that cultural practices, traditions and norms can play out differently in different situations, and the degrees and ways in which an individual has a sense of belonging to their culture will affect their perspective, the responses they consider appropriate, decisions, and ultimately Life Course [10]. Other authors have argued for an increased cross-cultural perspective to understanding the Life Course [35,36]. Many Life Course analyses have been done within a specific country, which may make the influence of culture less visible. In much of traditional Life Course theory, a male European view predominates[35]. Our analysis, done within a single country, Canada, highlights the importance of cultural differences between Indigenous Canadians and non-Indigenous Canadians, and although anecdotal, differences emerged in our data between new Canadians with different cultural backgrounds, and the dominant responses from euro-Canadians as well. Life Course theory does not explicitly address individual or group identifiers such as gender, race, religion, or culture which have cross cutting effects on all Life Course constructs. When considering Life Course analysis of the COVID-19 pandemic, culture might be particularly important given that the pandemic affected all areas of the globe, with the wide cultural variation that implies. We propose adding “Culture” to the Life Course principles, or alternatively explicitly including a cultural dimension to existing principles, as has sometimes been done, including linked lives, and time and place, to ensure that the impact of an individual’s experience of culture on Life Course is not lost.

### Implications for research and policy

The program and policy implications of a Life Course understanding of the impacts of COVID-19 are significant. First, a deeper understanding of the disruptions to transitions and social pathways can help us understand short term impacts of the pandemic as we are seeing them play out, including educational gaps, mental health struggles, substance use and youth unemployment [1,2,4,9].

Second, an awareness of turning points experienced during the pandemic can help us anticipate services that will be needed for certain particularly vulnerable groups in the short and medium term. These could include specific training and support for child and youth workers in school systems, child and family services, and youth mental health agencies as they try to support children and youth whose trajectories have been disrupted, with meaningful impacts on their socialization skills, physical and mental health, learning, development and identity formation. Attention to aspects of life course development such as the development of agency, impact of linked lives (specifically healthy relationships with others) should be considered. Marking transitions, even if retroactively, should be contemplated for children and youth, as well as adults, who have not been able to mark important transitions with ceremony or who have had these delayed.

Third, an awareness of the pervasiveness of the impacts of the pandemic across geographic locations, age groups and developmental stages can remind policy makers to anticipate longer term impacts of the pandemic as they begin to emerge. These will include the life-long impacts of adverse childhood experiences [2,9], as well as dysfunctional or missed transitions. Young adults who have not been able to transition to independent roles, due to deferred or online higher education, or through lack of employment opportunities, may need specific mentorship and support programs to retroactively ensure these transitions happen in a good way. Educators, as well as health and social service providers, may need additional training and support to help children and families who have experienced increased adversity during the pandemic to heal and develop healthy relationships. Substantial long term resource allocation may be required, which may challenge policy makers faced by competing demands, including those presented by the equally important crisis of climate change.

Fourth, understanding the importance of agency and identity as core constructs to good physical, social and emotional health, and how these have been disrupted, may allow for specific programming particularly though not exclusively for children and youth. Considering our data in light of the damaging impacts of the COVID-19 pandemic on individual agency and the complex process of identity formation may be helpful in understanding some of the long-term consequences of the pandemic and envisioning additional educational, mental health, job creation, and other supports that need to be put in place to help people work their way towards physically, socially and emotionally healthier life trajectories. Community agency is also important, and community development strategies inspired by models that have already demonstrate success [37,38] could be adapted to the ongoing pandemic context Ensuring that people and communities have meaningful sense of agency and an ability to positively improve their lives and that of those around them can provide an important resource to enhancing our recovery from the current pandemic, and our preparedness for other challenges such as future pandemics, natural disasters or social unrest.

## Limitations

Our study, as those cited above, is limited by the timeframe with which we can currently examine the Life Course impacts of the pandemic. Many more findings will emerge concerning the Life Course implications that have yet to unfold. Additionally, our study has some methodological limitations. Spryng.io micronarrative collection was both opportunistic in the first instance, and then intentional. This combination carries a risk of bias. Approximately 6% of the population of KFL&A identifies as Indigenous, and approximately 7% as “visible minority” [17]. Our sample overrepresented Indigenous People, and underrepresented visible minorities and men. As discussed in the introduction, the region where data were collected was relatively spared during the first waves of the COVID-19 pandemic, (this has since changed [39]), and it is likely that some of the implications of the pandemic on Life Course constructs could have been illustrated differently and more profoundly in areas which were more acutely affected by illness and death.

## Conclusions

Overall, an understanding of the implications of COVID-19 pandemic through a Life Course lens is a reminder to service providers and policy makers that people will continue to experience the sequelae of the pandemic for decades to come. Acutely, this understanding must inform the real time decisions policy makers continue to make about public health countermeasures and special programming related to the ongoing pandemic and society’s recovery. For some, this fallout will be minimal, but for others it will radically alter their life course in ways that will require long term creativity and care to support. Life Course theory can allow us to engage with the complexities of the impacts of the pandemic and to develop a more nuanced understanding of the implications of major disruptions, enabling us to come together as communities to imagine how to truly be “all in this together” for the long-term response to and recovery from COVID-19 and other pandemics which are sure to come.

## Supporting information

S1 File (DOCX)
