# Supplementary material for: Directed content analysis: A life course approach to understanding the impacts of the COVID-19 pandemic with implications for public health and social service policy
Source: PLoS One. 2022 Dec 16;17(12):e0278240. doi: 10.1371/journal.pone.0278240 (PMC9757550; doi:10.1371/journal.pone.0278240)
Supplement: S1 File — (DOCX) [file pone.0278240.s001.docx]

**Impact of COVID-19 Interview Guide**

**Introduction:**

Did you have a chance to read the Letter of Information and Consent form?

Do you have any questions about the study?

*If no more questions:*

- Do you consent to participating in this study?
- Do you consent for this interview being recorded?
- Do you consent to the use of anonymous quotes from this interview for publication and presentation purposes?

I will start recording now. In order to have your consent on record, I will repeat the consent questions.

*start recording, repeat three questions above*

Thank you for agreeing to participate in this study. We are interested in examining the impact of the COVID-19 pandemic on the mental health and well-being of people in your community, particularly children, youth and families, as well as Indigenous people. We are also interested in exploring the impact of the pandemic on household conflict. Finally, want to know about your clients’ experiences accessing health and social services during the pandemic.

**Demographic Questionnaire:**

To help us better understand your answers, I would like to collect some information about your organization and yourself.

1. What is title of your organization? TY
2. Please identify populations serviced by your organization. I will read you the options.
   1. Children
   2. Youth
   3. Women
   4. Men
   5. LGBTQ* population
   6. Indigenous people
   7. Newcomers
   8. Visible minorities
   9. People living in poverty
   10. People experiencing domestic violence
   11. People experiencing homelessness
   12. People experiencing mental health conditions
   13. People with physical health conditions
   14. People with intellectual or developmental disabilities
   15. People using substances
   16. All of the above
   17. Others: Please identify
3. What type of area does your organization predominantly serve? I read you the options:
   1. Urban
   2. Rural
   3. Both
4. Before the COVID-19 pandemic, did you offer any of the following services? Please select from the following options. To what extent? (none, a little, a lot)
   1. Phone services as emergency services
   2. Phone services as routine client services
   3. Videoconferencing
   4. Texting or other messaging with clients
   5. Emailing clients
   6. Drop in services
   7. Home visits
   8. Services/meetings with clients in public spaces
   9. Other (please list):
5. Since the beginning of the COVID-19 pandemic, have you been offering any of the following services? To what extent? (none, a little, a lot)
   1. Phone services as emergency services
   2. Phone services as routine client services
   3. Videoconferencing
   4. Texting or other messaging with clients
   5. Emailing clients
   6. Drop in services
   7. Home visits
   8. Services/meetings with clients in public spaces
   9. Other (please list):
6. And now a few questions about yourself
   1. The number of years you have worked at your organization
   2. Your role within your organization (administrator, service provider, etc). This information will be kept confidential.
7. **Overview**

Tell me the impact that the COVID-19 had on the individuals and families you serve, particularly as pertains to their mental health and well-being, and family conflict.

*Prompt*

*Can you give me another (a different) example of the impacts of COVID-19 on your clients?*

1. **Impact on Mental Health**

I would like to hear about the impact of the pandemic on the mental health of your clients.

Did you notice a specific impact on the mental health of your clients in general? *Can you tell me more about that?*

Now I would like to hear about specific population groups.

Did you notice a specific impact on the mental health of children or youth? *Can you tell me more about that?*

Did you notice a specific impact on the mental health of Indigenous clients? *Can you tell me more about that?*

Did you notice an impact on other specific groups that you work with? *Can you tell me more about that? Consider people living in poverty, people experiencing homelessness, LGBTQ population, newcomers, people using substances, other groups*

*Prompts*

*Any other impacts you can think of?*

*(Guide them to give an example of a different type of story (positive ->negative, negative -> positive)*

1. **Impact on Family/Partner conflict and violence**

Do you think that there was a change in the levels of interpersonal or family conflict experienced by your clients during the pandemic? Can you talk about the changes you saw?

*If yes, ask:*

- If you noticed an increase in conflict, were you able to address this at all? How?
- What were barriers or facilitators to addressing these issues in the context of the pandemic?
- Were there specific barriers or facilitators that presented differently for specific groups?

*If no, move to next question:*

Were there children or youth in your service whom you worried about? If so, why? Were you able to reach out to them? How? Which children or youth do you think were particularly affected by the pandemic?

Are there ways in which you think that your Indigenous clients might have been affected differently, or in particular ways, with respect to family conflict in the context of the pandemic?

1. **Impact on access to services**

Did you notice any trends in terms of who did or did not access your services during the pandemic? What are your thoughts on those trends?

Did you see an increase or decrease in service use? How did that change over the last number of months since the pandemic began?

Were these changes in service use different for different groups? *Consider children, youth, families, Indigenous people, or other specific groups.*

1. **Service adaptations**

How did your service delivery change over the course of the pandemic? (eg: were your services primarily virtual, did you use video conferencing at all? Did you use a phone? Did you have to stop offering certain services?)

If relevant to your organization, how were services and programs provided or run by volunteers affected?

What worked well? What benefits did you see in these service delivery changes?

What were the limitations *(consider privacy, confidentiality, etc)?*

Do you think these adaptations affected people experiencing mental health issues? *How?*

Do you think they affected children, youth and families? *How?*

Do you think they affected Indigenous people? *How?*

Were there specific groups that were most vulnerable to the impacts of the changes in service delivery? *Consider people living in poverty, people experiencing homelessness, LGBTQ population, newcomers, people using substances, other groups.*

1. **Ideas for the future**

If we continue to experience the impacts of the COVID-19 pandemic for the next 6-18 months, how do you think services need to adapt or changed to be able to offer best services to:

- People experiencing mental health issues?
- Children, youth and families?
- People experiencing family violence?
- Indigenous people?
- Are there any other groups that would benefit from specific service adaptations that you can think of?

1. **Closing**

Thank you for your time. We are just about done.

Can you tell me what you think the most important thing that we talked about in this interview is for you? *Why?*

Your answers will be very helpful to us in understanding the impact of COVID-19 pandemic on mental health and family conflict as well as on service delivery. We hope our findings will help improve service delivery over the course of the pandemic.

Before we close, is there anything else you would like to share regarding the impact of COVID-19 on your clients and service delivery?
